# Moral sensitivity of nursing students. Adaptation and validation of the moral sensitivity questionnaire in Spain

**DOI:** 10.1371/journal.pone.0270049

**Published:** 2022-06-16

**Authors:** Maria Francisca Jiménez-Herrera, Isabel Font-Jimenez, Leticia Bazo-Hernández, Juan Roldán-Merino, Ainoa Biurrun-Garrido, Barbara Hurtado-Pardos

**Affiliations:** 1 Universitat Rovira I Virgili, Tarragona, Spain; 2 Universidad Europea de Madrid Campus de Villaviciosa de Odón, Madrid, Spain; 3 Campus Docent, Sant Joan de Déu - Fundació Privada, School of Nursing, University of Barcelona, Barcelona, Spain; 4 Research Group GIES (Grupo de investigación en Enfermería, Educación y Sociedad), Barcelona, Spain; 5 Research Group GEIMAC (Consolidated Group 2017-1681: Group of Studies of Invarianza of the Instruments of Measurement and Analysis of Change in the Social and Health Areas), Barcelona, Spain; 6 Member Research Group GRIN (Grupo consolidado de recerca Infermeria, SRG:664), Barcelona, Spain; Murcia University, Spain, SPAIN

## Abstract

Ethical sensitivity is a requirement for people care as well as for decision-making in everyday practice. The aim is to present an adaptation and transcultural validation -in Spanish- of the Moral Sensitivity Questionnaire by Lützén et al. in Spain. In addition to that, we provide a practical implementation analysing the degree of moral sensitivity of nursing students. The data used for data collection were moral Sensitivity Questionnaire, socio-demographic data and a self-report questionnaire. The psychometric properties of the questionnaire were assessed, including validity and reliability. Fit indices of the overall model were computed. The fit indices of the Confirmatory Factor Analysis (CFA) indicate a poor fit, although the Exploratory Factor Analysis (EFA) revealed two dimensions that show a better fit of its indices. Women and those women with more experience in the clinical setting have a higher mean score, as well as those who study in centers where the strategic lines are the humanization of care. Female nursing students with more experience in the clinical setting and with more educational training present higher sensitivity indexes, as well as those who study in centers where the strategic lines are the humanization of care. The findings confirm that the Lützén et al. questionnaire is multidimensional. In the Spanish sample, it was necessary to group the three initial factors into two: sense of moral burden and moral strength—grouping the moral responsibility items into the above items to make the instrument more resilient.

## Introduction

Advances in health sciences and the technification of health care have led to the emergence of ethical problems [1]. In order to deal with them, it is essential that health care professionals must have high ethical skills. Authors such as Fry and Johnstone [2] highlighted the relevance of considering ethical practice when a quality care is provided, and therefore, they identified ethics as a necessary competence in nursing professionals.

Nowadays, bioethics is a compulsory subject in health care curricula [3]. In the case of the nursing profession, many articles highlight the importance of training nursing students in ethical aspects by carrying out studies to measure: the student’s degree of sensitivity [4–7]; and the acquisition of ethical skills [8, 9]—part where concepts such as ethical sensitivity and moral sensitivity are introduced. These last terms are used interchangeably, even though—in reality—they present some nuances.

Authors such as Rest [10] explained that decision-making process in nursing is facilitated by ethical sensitivity as a key element. Thus, dealing with ethical problems requires that healthcare professionals have a certain grade of ethical sensitivity and their ability to identify ethical problems. Healthcare professionals who exhibit ethical sensitivity are most capable of assessing the feelings and responses of others, as well as being aware of possible courses of action. Similarly, Weaver [11] defined ethical sensitivity as " the capacity to decide with intelligence and compassion, given uncertainty in a care situation, drawing as needed on a critical understanding of codes for ethical conduct, clinical experience, academic learning and self-knowledge, with an additional ability to anticipate consequences". On the other hand, Lützén [12] examined the concept of moral sensitivity and defined it as the ability to perceive moral conflict acknowledging ethical consequences of the decisions made and showing a wide understanding of patient’s vulnerable situation. Hence, it is possible to understand moral sensitivity according to different dimensions, but essentially, from a personal perspective, it refers to the person’s capacity to be aware that their actions affect or may affect others [12].

Therefore, it can be asserted that moral sensitivity is a mediator of the relationship between motivation and moral reasoning that, at the same time, allows the development and the promotion of the advancement and maturation of moral reasoning [13]. Furthermore, moral sensitivity is changeable and unstable, reason why some authors have delved into the content and meaning of epistemological development [14–16].

However, nursing professionals are often faced with serious situations that require them to be sensitive to moral issues in order to be able to make the right decisions [17]. Moral sensitivity allows a patient-nurse relationship focusing on confidence and the availability to respond to individual needs; this way, they promote the autonomy of the patient to protect their vulnerability [18].

As far as the nursing profession is concerned, the most outstanding studies on nurses’ ethical sensitivity come from the 1980s [18]—with Lützén as one of the pioneering authors on the subject. Lützén [19] described moral sensitivity as a process in which nurses examine, analyze, justify, choose and evaluate decisions in ethical situations. Later, in 1994, together with Nordin and Brolin, they developed the Moral Sensitivity Questionnaire (MSQ)—questionnaire which years later was modified and widely used along with other similar questionnaires [20].

Given that currently health professionals are expected to show an outstanding degree of ethical skills, different tools have been sought for their assessment [21]. Ethical sensitivity is the required ability to recognize ethical problems, and therefore, necessary to solve them. The MSQ is the most widely used tool for assessing ethical sensitivity and it has been translated and adapted to different languages and cultures [9, 22–24].

In addition, one of the pioneering authors in measuring moral sensitivity in nursing students modified the original MSQ to facilitate its quantitative measurement and defined it as the Modified Moral Sensitivity Questionnaire for Nursing Students (MMSQ-SN).

To our knowledge, in the Spanish context there are no validated tools in Spanish to assess moral sensitivity in nursing students; for this reason, the objectives of the study were to provide a cross-culturally adaptation and validate the Moral Sensitivity Questionnaire of Lützén et al. for students–from the original English version to Spanish—and examine its reliability and validity. In addition, we sought to determine in nursing students in Catalonia, which is the degree of moral sensitivity and its relationship with the sociodemographic characteristics.

## Methods

### Design

It was used a methodological approach with two phases. First we carry out a translation, adaptation and cross-cultural validation of the questionnaire, and then it is followed by a descriptive and correlational study.

### Participants and setting

The sample of the study consisted of 751 nursing students enrolled during the 2019 and 2020 academic year from four university campus located on the autonomous community of Catalonia (Spain). We used a non-probabilistic convenience as a sampling technique, in which the inclusion criteria of the study were defined as follows: to be a student of the degree in nursing in its second, third or fourth year; to have completed a nursing internship; and to have provided an informed consent to the students about their involvement in the study. The exclusion criterion was to not be present in the classroom on the day the questionnaires were collected.

To estimate the sample size we have considered Comrey and Lee [25] recommendations for validation studies, who consider that a very good sample has between 500 and 1000 participants.

### Variables and sources of information

The MSQ-SPV was provided to nursing students jointly with an ad-hoc form to fill in variables about sociodemographic and occupational aspects of the nursing students.

The MSQ-SPV questionnaire is a self-administered questionnaire; it has nine items or assumptions with six possible answers, where the value 1 means absolute disagreement with the statement and 6 complete agreements. These nine items are grouped into three factors: Moral strength (3 items); Sense of moral burden (4 items); and Moral responsibility (2 items) (Table 1). The minimum score is 9 points, and the maximum score is 54. Thus, a higher the score on the questionnaire is directly related to a higher measure of participant’s moral sensitivity.

**Table 1:** Lexical and semantic equivalence of the MSQ-SPV.

English Version	Spanish Version
**Sense of moral burden**	
**Item 4**. My ability to sense the patient’s needs means that I do more than I have the strength for.	**Ítem 4**. Mi capacidad para percibir las necesidades del paciente me lleva a hacer más de lo que puedo asumir.
**Item 6**. II find it very difficult to deal with my feelings that are aroused when a patient is suffering.	**Ítem 6**. Es difícil gestionar mis emociones cuando un paciente está sufriendo.
**Item 7**. When caring for patients, I am always aware of the balance between the potential for doing good and the risk of causing harm to them.	**Ítem 7**. Cuando cuido de los pacientes siempre soy consciente de la posibilidad de hacerles un bien y del riesgo de causarles daño
**Item 8**. My ability to sense a patient’s needs means that I often find myself in situations in which I feel inadequate.	**Ítem 8**. Mi capacidad para percibir las necesidades del paciente conlleva que a menudo, me encuentre en situaciones en las que me siento incómodo.
**Moral strength**	
**Item 2**. My ability to sense the patient’s needs is always helpful in my work.	**Ítem 2**. Mi capacidad de percibir las necesidades del paciente siempre me ayuda en mi trabajo.
**Item 3**. I have a very good ability to feel how I should talk about difficult things with the patient.	**Ítem 3**. Tengo muy buena capacidad para saber si a un paciente se le debe decir la verdad y cuando.
**Item 5**. I have a very good ability to sense when a patient is not receiving good care.	**Ítem 5**. Tengo muy buena capacidad para saber cuándo el paciente no está recibiendo un buen cuidado.
**Moral responsibility**	
**Item 1**. I always feel a responsibility for the patient receiving good care even if the resources are inadequate.	**Ítem 1**. Siempre siento la responsabilidad de asegurarme que los pacientes reciban un buen cuidado, aunque los recursos no sean los adecuados.
**Item 9**. It helps me to know what is good or bad for the patient when I can follow rules and regulations.	**Ítem 9**. Considero que cuando puedo trabajar de acuerdo con las normas y los reglamentos, raramente me resulta difícil saber lo que es bueno o malo para el paciente.

### Procedure

The study was carried out in two steps: First, we translated and adapted the English version of the questionnaire to the Spanish version. In order to do that, an independent committee of Spanish-English and English-Spanish with bilingual experts was created. The expert committee carried out the translation and back-translation procedure according to the Standards for Educational and Psychological Testing [26] and it was formed by: two female professors of ethics with advanced knowledge in English and Spanish languages; two female professors of nursing with clinical experience; and two female professors with expertise in psychometrics.

They began by making an individual and then a joint translation of the original MSQ questionnaire. There was consensus that the items of the Spanish questionnaire coincided with the original version. In order to adapt it to the Spanish cultural context, the term "to feel" was replaced by the term "to know" (*saber*) in item 3 and the term "balance" was replaced by the term "possibility" (*posibilidad*) in item 7.

In addition to the questionnaire, we collected other variables such as age, gender, university campus, whether they are working, the type of contract and their experience in the health care setting.

The lexical and semantic equivalence of the Spanish version of the Lützén et al. questionnaire (MSQ-SPV) is shown in Table 1.

The questionnaire was subjected to a pretest to detect possible comprehension difficulties and to estimate completion time. The time required to complete it ranged from 5 to 10 minutes. After asking the questions, it was concluded that the questionnaire’s format or content did not require any changes.

Second, we analyzed the psychometric properties of the MSQ-SPV and specifically we determined and the level of sensitivity of the nursing students. Each center planned a day to provide the questionnaire to the nursing students. All students were informed—verbally and through a written information sheet—of the purpose of the study to resolve any doubts about the study. The questionnaire and the informed consent were given to all those willingly agreed to be part of the study.

### Statistical analysis

In order to analyze psychometric properties of the questionnaire, it was performed a confirmatory factor analysis with a generalized least squares method. The objective of this analysis is to ascertain if the results of the questionnaire reproduce the three-dimensional structure that the original MSQ is based on. This method used in data analysis has similar properties as maximum likelihood method; however, multivariate normality considerations are less rigorous and it is mainly employed for ordinal items [27]. In this study, the following global fit indices were used: Normalized Chi-squared test defined as the quotient between the Chi-squared test statistic and the degrees of freedom (x2/df); CFI (Comparative Fit Index); GFI (Goodness-of-fit Index); AGFI (Adjusted Goodness-of-fit Index); BBNFI (Bentler Bonnet Normed Fit Index); BBNNFI (Bentler Bonnet Non-Normed Fit Index); RMSEA (Root Mean Standard Error of Approximation); SRMR (Standardized Root Mean Square Residual); and RMR (Root Mean Square Residual). The criterion adopted to consider a good global adjustment will be that of obtaining the following fitting values: normalized chi-squared ratio from 2 to 6 [28]; GFI, CFI, AGFI, BBNFI, BBNNFI > 0.95; and SRMR, RMSEA and RMR < 0.08 [29].

Regarding Questionnaire’s reliability it was analyzed by means of internal consistency with Cronbach’s alpha test which considered alpha of 0.70 as adequate value [30].

The homogeneity of the questionnaire’s corrected items was also estimated. To do that, we have obtained for each item of the questionnaire, the cross-correlations with the rest of the items. It was taken a correlation value of 0.20 as an admissible lower bound [31].

To assess the level of moral sensitivity, first we performed a normal distribution test to evaluate if the data follow a normal distribution using a Kolmogorov-Smirnov test. Since the data were not normally distributed we used non-parametric statistical tests. For the data analysis, descriptive statistics (mean, deviation, frequency, and percentages according to the type of variable) were used. To analyze the relationship between the degree of moral sensitivity with sociodemographic- and work-variables of the students, the Spearman correlation test, the Kruskal-Wallis test and the Mann-Whitney U test were used.

The data analysis has been carried out with the statistical software SPSS 17 for Windows (SPSS Institute, Chicago, IL, USA, 2008); CFA was performed using EQS 6.1 for Windows, (Multivariate Software, Inc., 2006) and the Factor Analysis program for Horn’s Parallel Analysis were used.

### Ethical consideration

The study was approved by the Clinical Investigation Ethics Committee of the Sant Joan de Déu Foundation with CEIC research code PIC-43-19. Legal regulations on the confidentiality of personal data were followed for the study, just as good research practices described in the Declaration of Helsinki.

All nursing students were informed about the willingness of their participation in the study and the absence of any compensation as a reward for taking part of it. In particular, students received—orally and in written form—information about the treatment and the confidentiality of the data.

They were asked for a signed informed consent to take part of the study, emphasizing that data will only be used for the study’s purposes. Authorization was requested from the MSQ author to provide a translated and cultural adapted version of the questionnaire in Spanish.

## Results

The sample of the study consisted of 751 nursing students. The mean age was 22.9 (±5.5) years, with 86.0% (n = 646) being female. A 52.9% (n = 397) reported to be currently working and 60.2% (n = 239) of these had temporary employment. More than half of the students—63.0% (n = 473)—were enrolled in the morning shift (Table 2).

**Table 2:** Sociodemographic characteristics of the study population.

	n	%
Age (SD)	22.9 (SD 5.5)	
Gender		
Female	646	86.0
Male	105	14.0
Academic year		
Second	270	36.0
Third	326	43.4
Fourth	155	20.6
Center		
Campus SJD (UB)	447	59.5
Terres de l’Ebre (URV)	132	17.6
Baix Penedés (URV)	85	11.3
Campus Catalunya (URV)	87	11.6
Study schedule		
Morning	473	63.0
Afternoon	241	32.1
Morning and afternoon	37	4.9
Currently employed		
Yes	397	52.9
Not	354	47.1
Previous work experience in healthcare		
Yes	259	65.2
Not	138	34.8
Type of contract		
Permanent employment	158	39.8
Temporary employment	239	60.2

### Reliability and validity of the moral sensitivity questionnaire—Spanish version

The MSQ-SPV obtained a Cronbach’s value based on standardized items of 0.649; the 9 items obtained values between 0.609 and 0.643 (Table 3).

**Table 3:** Cronbach’s α and moral sensitivity questionnaire index for each item.

	Cronbach’s a if the item is eliminated	Mean score if the item is eliminated	Variance if the item is eliminated	Mean (SD)
Item 1	.637	36.89	20.315	5,6 (0,6)
Item 2	.616	37.21	19.369	5,3 (0,7)
Item 3	.621	38.08	18.816	4,4 (0,9)
Item 4	.593	38.14	17.257	4.3 (1,0)
Item 5	.622	37.41	19.263	5,1 (0,8)
Item 6	.643	38.26	17.581	4.2 (1,3)
Item 7	.628	37.22	19.689	5,2 (0,7)
Item 8	.623	38.63	17.000	3,8 (1,3)
Item 9	.609	38.31	16.826	4,2 (1,2)

Mean scale 42,51 (SD 4,7); variance scale 22,565; Cronbach’s α = 0.649.

#### Confirmatory Factor Analysis (CFA)

A Confirmatory Factor Analysis with a three dimensional model was proposed, a model which has identical structure of the original version of the questionnaire. Regarding the results of the CFA, a significant chi-square value was obtained [χ2 = 247.709; p < .001; n = 751]; CFI = .827; BBNFI = .806; BBNNFI = .741; GFI = .973; AGFI = .949; SRMR = 0.088; RMSEA = .087 and RMR = .069. For all indices, the results obtained indicate a poor fit; we obtain a value for all indices below and near to .095, as reflected in CFI, BBNFI and BBNNFI indices. This result similar to what happens for the error values (RMSEA, SRMR, RMR), which are equal to or below .08.

As the fit of the CFA was not as expected of the Spanish population, we decided to perform an Exploratory Factor Analysis (EFA) to test which model should be expected from it. For this purpose, a classical implementation method of Horn’s Parallel Analysis [32] was used. This method provides better results than conventional methods as it identifies the right number of dimensions [33, 34]. The item scores were considered as ordinal categorical variables and the EFA was fitted into the inter-item polychoric correlation matrix [35]. The coefficients in the correlation matrix for the items were examined considering coefficients over 0.30 as significant. As a fit function an unweighted robust least squared was chosen, with mean and variance corrected fit statistics [36]. Robust Promin rotation was used to rotate factors [35].

The EFA revealed 2 dimensions explaining variance in 45.9%. Table 4 shows the loading matrix related to the EFA solution.

**Table 4:** Loading matrix related to the exploratory factor analysis solution.

Item	Factor 1	Factor 2
1. Feel responsibility	0.711	
2. Ability to sense	0.532	
3. Ability to talk	0.437	
4. Sense need	0.353	
5. Sense not good care	0.493	
6. Suffering		0.372
7. Balance between good and harm	0.548	
8. Feel inadequate		0.882
9. Rules and regulations		0.352

Table 5 depicts goodness-of-fit indices for the two-factor model analyzed in the sample of the Spanish student population. All the indices analyzed showed that the model provides a good fit.

**Table 5:** Indices of goodness of fit of the exploratory factor analysis to the model.

INDEX	VALUE	95% confidence interval
CFI	0.980	0.969–0.998
GFI	0.991	0.988–0.996
AGFI	0.982	0.978–0.992
RMSEA	0.045	0.016–0.053
Goodness of fit test	χ2 = 48.441; gl = 19; p < 0.001	
Reason for fit	χ2 / gl = 2.54	

**CFI**: Comparative Fit Index. **GFI**: Goodness of Fit Index. **AGFI**: Adjusted Goodness of Fit Index. **RMSEA**: Root Mean Standard Error of Approximation

### Levels of exposure to moral sensitivity and sociodemographic characteristics

The global mean score of the questionnaire was 42.5 (SD 4.7); the value of the score ranges from 11 as a minimum value to 54 as a maximum value.

Table 6 presents comparative values of the mean scores of the questionnaire for each of its dimensions and also for the total score in relation to some characteristics of the students. For this analysis, the three dimensions identified in the original version of the questionnaire were considered. With respect to age, only a positive and statistically significant correlation (rho = .177; p < .001) was found with factor 2 (moral strength) in the sense that the older the student, the greater the moral strength. A statistically significant difference was also observed with respect to gender. Women presented a higher mean score for F1 (sense of moral burden) (mean 17.9, SD 2.8, p = .003) and for the total score of the questionnaire (mean 42.6, SD 4.7, p = .024). It was also observed that an increase in the year of study correlated with higher sensitivity, which is statistically significant for the total and for each of the dimensions of the questionnaire. In addition, students belonging to one of the universities participants (whose principles are based in moral Christianity) had higher scores compared to the rest of the students enrolled in the other universities. These scores were also statistically significant in F1 (mean 18.0, SD 2.9, p = .027), F2 (mean 15.1, SD 1.7, p < .001) and for the total questionnaire (mean 43.0, SD 4.9, p < .001). The shift of the study had any relation to the degree of moral sensitivity declared by the students. However, students who declared to be working and to have experience in the health field presented a higher mean score in F2 (moral strength), which is statistically significant (p = .001 and p = .0001 respectively). The type of contract did not influence the level of moral sensitivity declared by the students.

**Table 6:** Levels of exposure to sensitivity moral and sociodemographic characteristics.

Variables	Sense of moral burden			Moral Strength			Moral responsibility			Total Sensibility Moral		
	Mean	SD	p	Mean	SD	p	Mean	SD		Mean	SD	P
Age	Rho = -.003; p = .945^1^			Rho = .177; p = .0001^1^			Rho = .009; p = .810^1^			Rho = .055; p = .133^1^		
**Gender**												
Female	17.9	2.8	**.003** ^2^	14.8	1.8	.604^2^	9.8	1.5	.525^2^	42.6	4.7	**.024** ^2^
Male	17,0	3.0		14.9	1.5		9.7	1.6		41.7	4.8	
**Academic year**												
Second	17.8	2.8	**.015** ^3^	14.7	1.7	**.0001** ^3^	9.8	1.4	**.008** ^3^	42.4	4.4	**.0001** ^3^
Third	17.5	2.7		14.6	1.9		9.6	1.5		41.8	4.6	
Fourth	18.3	3.0		15.4	1.5		10.0	1.5		43.9	5.0	
**Center**												
Campus SJD (Universitat de Barcelona)	18.0	2.9	**.027** ^3^	15.1	1.7	**.0001** ^3^	9.8	1.5	.070^3^	43.0	4.9	**.0001** ^3^
Terres de l’Ebre (URV)	17.5	2.7		14.4	1.9		9.6	1.6		41.6	4.6	
Baix Penedes (URV)	17.1	2.5		14.4	1.7		9.6	1.4		41.2	3.9	
Campus Catalunya (URV)	17.6	2.6		14.5	1.9		10.1	1.3		42.3	4.1	
**Study schedule**												
Morning	17.7	2.8	.293^3^	14.7	1.8	.125^3^	9.7	1.5	.434^3^	42.2	4.7	.098^3^
Afternoon	18.1	2.7		15.0	1.5		9.6	1.3		43.1	4.2	
Morning and afternoon	17.6	2.6		14.6	1.6		10.0	1.4		42.3	4.1	
**Currently employed**												
Yes	17.7	2.9	.567^2^	15.0	1.7	**.001** ^2^	9.8	1.5	.820^2^	42.6	5.0	.431^2^
Not	17.8	2.7		14.6	1.8		9.8	1.4		42.3	4.4	
**Previous work experience in healthcare**												
Yes	17.9	3.0	.087^2^	15.3	1.7	**.0001** ^2^	9.8	1.6	.197^2^	43.1	5.1	**.002** ^2^
Not	17.4	2.9		14.5	1.6		9.7	1.4		41.7	4.5	
**Type of contract**												
Permanent employment	17.9	2.8	.597^2^	15.0	1.7	.949^2^	9.8	1.5	.803^2^	42.9	4.6	.746^2^
Temporary employment	17.7	3.0		15.0	1.8		9.7	1.6		42.4	5.2	

^1^: Spearman correlation test;

^2^: Mann-Whitney U test

^3^: Kruskal-Wallis test

## Discussion

On one hand, the main objective was to validate the MSQ-SPV in terms of reliability and construct validity, and on the other hand, to know the degree of moral sensitivity of the students concerning sociodemographic and occupational characteristics.

The MSQ was developed to assess moral sensitivity through grouped in three main factors: moral responsibility, moral strength and the sense of moral burden. The meaning of moral burden can be interpreted as the negative valence of ethical sensitivity. If the sense of moral burden is very high, it can cause moral stress in nurses—this result agrees with other studies that affirm that nurses with high moral sensitivity suffer moral distress [37].

Regarding to construct validity and terms of internal consistency, the results obtained show that the Spanish version of the questionnaire has weaker psychometric properties than those obtained in the original version [12]. The CFA revealed a poor fit of the questionnaire structure with three dimensions and 9 items. Not all the values obtained from the indices and used for goodness-of-fit and factorial validity were good enough, which proves the poor fit of the model.

Some values of the model indices were acceptable, such as the GFI, AGFI and CFI, which are either above .095 or approximately this value. Similar to that happens with the values of the errors (SRMR, RMR, RMSEA), which are equal to or below .08 [29, 38, 39]. The values obtained for some indicators such as the CFI or RMSEA stand out. These last referents are some of the bests to show the suitability of a model, as are not related to the size of the sample—this means that it supports the viability of the measurement model [28].

Regarding the internal consistency of the questionnaire, it was shown reliability values close to .70—always taken into account that when a measurement instrument is developed a reliability equal to or higher than 0.70 is the minimum acceptable generally suggested [28, 40]. These values were slightly lower than those reported in the Chinese, the English and the Korean versions [23, 24, 41].

The EFA revealed that the model with two-factor is the best fit. These results are similar to those found in the Chinese version in which a two-factor model was also identified [23].

The percentage of explained variance by this model could be understood as low, as it is below 60% [42]. Nevertheless, currently it is not recommended to keep an interpretation of explained variance based only on the indicator of identified factors, and it should be incorporated methods of Parallel Analysis—selection of common components or factors that present eigenvalues higher than those expected by chance—or the RMSEA fit indicator [43] which provide the number of factors. Both statistical methods (parallel analysis and RMSEA) have been used in the Spanish sample to estimate the appropriate number of factors.

Despite these results, further studies are necessary to corroborate the factor structure of the MSQ-SPV to verify which of the two models would be the most appropriate for assessing the level of moral sensitivity—in contrast to other authors who confirm that the instrument is in accordance with a theoretical conceptualization of moral sensitivity [6].

The sense of moral burden seems to be negative aspect of moral sensitivity which shows that those personnel who are not adequately trained to deal with morally disturbing situations are at greater risk of suffering moral stress. As in other studies [18, 41, 44], the participants were mostly women and this dimension affects the female gender more. Women obtained higher scores in the degree of moral sensitivity [45]; and this is relevant since it could be a definitive element in moral sensitivity. On the same line, some authors such as Lützén [46] point out that there is a difference between genders in moral sensitivity. In addition, Tas Arslan [47] mentions in his study that female nurses as opposed to male nurses, tend to have greater moral sensitivity and more holistic approaches. Likewise, this could be related to the ethics of care theory proposed by Carol Gilligan [48].

It can be derived from the results that second year students have just received training in ethics of care, and this may influence a better result by comparing it to the results of senior and older students—who have already had the vast majority of training in the clinical practice setting and have lived real experiences [49]. This could coincide with the results obtained by Park and Kjervik [50] who observed that moral sensitivity increases by means of ethical education; meaning that senior students of nursing showed higher scores than first year students. These authors affirm that as there is a greater training, education, reflection and discussion about ethical issues in professionals and nursing students, their degree of moral sensitivity is being increased in caring relationships.

It should be noted that the University campus which shows a greater levels of moral sensitivity in this research, belongs to a religious order whose strategic line is the humanization of care, which makes this philosophy more evident in comparison with secular institutions [51].

### Limitations

There are several limitations in this study which should be considered. Firstly, nursing students were selected by non-probabilistic convenience sampling and freely participated in the study; meaning that it could have been a selection bias. Nevertheless, in this research has participated a large number of students from universities in different locations and the profile of these students is similar to that of the rest of the students in the Spanish population; therefore, it is possible to generalize these results. Secondly, the overall reliability of the questionnaire found was slightly lower than expected and hence, the results should be interpreted with caution.

Nevertheless, temporal stability could also not be analyzed due to the type of questions that make up the questionnaire, as it may induce respondents to reflect on the subject. This reflection could in turn generate new attitudes towards the topic and consequently, cause inconsistencies in the answers between the two tests. Another limitation is that "sensitivity to change" was not analyzed. However, this could be studied in future post-intervention or longitudinal studies.

## Conclusions

Ethical sensitivity is a key aspect of the ethical decision-making process; however, the meaning of moral burden can be interpreted as the negative valence of ethical sensitivity. Therefore, this kind of instrument are important when educate nurses, to notice their progression in the subject. The results of this study show that female nursing students with more experience in the clinical setting and with more education present higher sensitivity indexes, as well as those who study in centers where the strategic lines are the humanization of care.

In addition, the findings confirm that the Lützén questionnaire is multidimensional. In the Spanish sample, it was necessary to group the three initial factors into two: moral strength and sense of moral burden–grouping the moral responsibility items into the above items to make the instrument more resilient.

## Supporting information

S1 File (XLSX)
